# 

**Published:** 1898-01

**Authors:** 


### BOOKS RECEIVED.

Diseases of the Eye. By Edward Nettleship, F. R. C. S., Ophthalmic Surgeon at St. Thomas' Hospital, London ; Surgeon to the Royal London (Moorfields) Ophthalmic Hospital. Revised and edited by W. T. Holmes Spicer, M. A., M. B., F. R. C. S., Ophthalmic Surgeon to the Metropolitan Hospital and to the Victoria Hospital for Children. Fifth American from the sixth English edition. With a supplement on color blindness by William Thomson, M. D., Emeritus Professor of Ophthalmology in the Jefferson Medical College of Philadelphia. Handsome 12mo of 521 pages, with two colored plates and 161 engravings. Cloth, $2.25. Philadelphia and New York : Lea Brothers & Co., Publishers. 1897.

Biennial Report of the Department of Health of the City of Chicago. For the years 1895 and 1896. William R. Kerr, Commissioner of Health. Chicago : 1897.

Manual of Pathology, including Bacteriology, the Technique of Post mortems and Methods of Pathologic Research. By W. M. Late Coplin, M. D., Professor of Pathology and Bacteriology, Jefferson Medical College, and Pathologist to Jefferson Medical College Hospital and to the Philadelphia (Blockley) Hospital ; Bacteriologist to the Pennsylvania State Board of Health. Being a second edition of the author's lectures on pathology, rewritten and enlarged. Small octavo, pp. xxi.—638. With 268 illustrations, many of which are original. Price, $3.00. Philadelphia: P. Blakiston, Son & Co., 1012 Walnut street. 1897.

Report of the Commissioner of Education. For the year 1895-96. Volume II. Containing Part II. Washington: Government Printing Office. 1897.

The Care and Feeding of Children. A catechism for the use of mothers and children's nurses. By L. Emmett Holt, M. D., Professor of Diseases of Children in the New York Polyclinic; Attending Physician to the Babies' Hospital and the Nursery and Child's Hospital, New York. Second edition, revised and enlarged. Duodecimo, pp. 104. Price, cloth, 50 cents. New York: D. Appleton & Co. 1897.

Diseases of the Stomach. Their special pathology, diagnosis and treatment, with sections on anatomy, physiology, analysis of stomach contents, dietetics, surgery of the stomach, etc. In three parts. By John C. Hemmeter, M. B., M. D., Philos. D., Clinical Professor of Medicine at the Baltimore Medical College; Consultant to the Maryland General Hospital, etc. Royal octavo, pp. xvii.—788. With many original illustrations, a number of which are in colors and a lithograph frontispiece. Price, $6.00. Philadelphia: P. Blakiston, Son & Co., 1012 Walnut street. 1897.

A Clinical Text-Book of Surgical Diagnosis and Treatment. For practitioners and students of surgery and medicine. By J. W. Macdonald, M. D., Graduate in Medicine, of the University of Edinburgh; Licentiate of the Royal College of Surgeons, Edinburgh; Professor of the Practice of Surgery and of Clinical Surgery in Hamline University, Minneapolis, etc. Royal octavo, pp. 798. With 328 illustrations. Price, cloth, $5.00; half morocco, $6.00, net. Philadelphia: W. B. Saunders, 925 Walnut street. 1898.

Twelfth Annual Report of the State Board of Health and Vital Statistics of the Commonwealth of Pennsylvania. Volumes I. and II. 1896.

Transactions of the Colorado State Medical Society. Twenty-seventh Annual Convention. By-laws and list of members. Denver, June, 1897.

An Epitomé of the History of Medicine. By Roswell Park, A. M., M. D., Professor of Surgery in the Medical Department of the University of Buffalo, etc. Illustrated with portraits and other engravings. One volume, royal octavo, pages xiv.—348. Extra cloth, beveled edges, $2.00, net. Philadelphia: The F. A. Davis Co., Publishers, 1914 and 1916 Cherry street; New York: 117 W. Forty-second street; Chicago: 9 Lakeside Building.

Clinical Methods, being an Introduction to the Practical Study of Medicine. By Robert Hutchison, M. D., M. R. C. P., Demonstrator of Physiology in London Hospital Medical College, and Harry Rainy, F. R. C. P. P., F. R. S. E., University Tutor in Clinical Medicine, Royal Infirmary, Edinburgh. Handsome 12mo, 562 pages, 137 engravings and 8 colored plates. Cloth, $3. Philadelphia and New York: Lea Brothers & Co., Publishers.

